# Selenium‐Doped Nanoheterojunctions for Highly Efficient Cancer Radiosensitization

**DOI:** 10.1002/advs.202402039

**Published:** 2024-06-03

**Authors:** Rui Qiao, Zhongwen Yuan, Meijin Yang, Zhiying Tang, Lizhen He, Tianfeng Chen

**Affiliations:** ^1^ College of Chemistry and Materials Science Department of Oncology of The First Affiliated Hospital Jinan University Guangzhou 510632 China

**Keywords:** nano‐heterojunction, physical and biochemical potentiation, radiosensitizers, redox imbalance, tumor radiotherapy

## Abstract

Exploring efficient and low‐toxicity radiosensitizers to break through the bottleneck of radiation tolerance, immunosuppression and poor prognosis remains one of the critical developmental challenges in radiotherapy. Nanoheterojunctions, due to their unique physicochemical properties, have demonstrated excellent radiosensitization effects in radiation energy deposition and in lifting tumor radiotherapy inhibition. Herein, they doped selenium (Se) into prussian blue (PB) to construct a nano‐heterojunction (Se@PB), which could promote the increase of Fe^2+^/Fe^3+^ ratio and conversion of Se to a high valence state with Se introduction. The Fe^2+^‐Se‐Fe^3+^ electron transfer chain accelerates the rate of electron transfer on the surface of the nanoparticles, which in turn endows it with efficient X‐ray energy transfer and electron transport capability, and enhances radiotherapy physical sensitivity. Furthermore, Se@PB induces glutathione (GSH) depletion and Fe^2+^ accumulation through pro‐Fenton reaction, thereby disturbs the redox balance in tumor cells and enhances biochemical sensitivity of radiotherapy. As an excellent radiosensitizer, Se@PB effectively enhances X‐ray induced mitochondrial dysfunction and DNA damage, thereby promotes cell apoptosis and synergistic cervical cancer radiotherapy. This study elucidates the radiosensitization mechanism of Se‐doped nanoheterojunction from the perspective of the electron transfer chain and biochemistry reaction, which provides an efficient and low‐toxic strategy in radiotherapy.

## Graphical abstract

For the development of highly effective and low‐toxic radiosensitizers, herein, the study develops a nano‐heterojunction (Se@PB) containing Fe^2+^‐Se‐Fe^3+^ electron transfer chains, which is inspired by the high safety of the trace elements selenium (Se) and Prussian blue (PB), and the unique activities of Se in strong electron contribution and its redox properties. The Se@PB nano‐heterojunction containing Fe^2+^‐Se‐Fe^3+^ chain exerts physical and biochemical radiosensitization by accelerating electron transfer and redox dyshomeostasis.

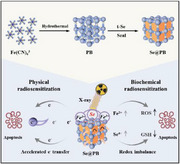

## 1 Introduction

Radiotherapy, as one of the traditional means of tumor treatment, has an irreplaceable position in contributing to clinical cancer cure due to its non‐invasive and localized direct treatment of lesions.^[^ ^1^ ^]^ Especially in the treatment of cervical cancer, ≈80% of patients need to receive radiotherapy.^[^ ^2^ ^]^ However, radiotherapy inevitably faces the bottleneck of radiation tolerance, immunosuppression, and poor prognosis due to the highly complex tumor microenvironment (TME).^[^ ^3^ ^]^ The external beam radiation therapy and brachytherapy are important treatment to manage the locoregional advanced cervical cancers, especially the brachytherapy, which can utilize invasive radioactive sources for tumor treatment.^[^ ^4^ ^]^ Owing to the poor deposition efficiency and energy transfer rate of radiation energy in tumor site, it is necessary to achieve therapeutic effects by increasing the radiation dose, which in turn damages the normal tissues surrounding the tumor.^[^ ^5^ ^]^ Therefore, the development of radiosensitizers for the purpose of reducing toxicity (lowering the radiation dose to patients) and increasing efficacy (improving the sensitivity of tumors to radiotherapy) is particularly urgent.^[^ ^6^ ^]^ In recent years, studies have found that radiotherapy sensitizers of nanomaterial not only enhance the deposition of X‐ray energy in tumor cells, but also emit secondary electrons under X‐ray radiation to improve the physical sensitivity of radiotherapy,^[^ ^7^ ^]^ such as inorganic metal nanoparticles,^[^ ^8^ ^]^ semiconductor heterojunctions,^[^ ^7^, ^9^ ^]^ metal‐organic frameworks,^[^ ^10^ ^]^ and nonmetallic nanomaterials.^[^ ^11^ ^]^ For instance, James C.L. Chow et al. explored and discovered an iron‐gold nanoparticle heterojunction to enhance radiation‐induced DNA damage,^[^ ^12^ ^]^ suggesting the important potential of heterojunction as radiosensitizer in radiotherapy. More importantly, these nanomaterials improve the utilization of radiation energy through enhancing electron transfer, thereby achieving physical sensitization of radiation therapy.^[^ ^13^ ^]^ However, the development of radiosensitizers that solely focus on physical sensitization to enhance cellular oxidative damage, while ignore a series of changes in TME caused by radiation, will lead to the occurrence of radiotherapy tolerance and resistance. For example, tumor cells exposed to radiotherapy will lead to the up‐regulation of DNA repair proteins, the enhancement of antioxidant system, and immunosuppressive environment.^[^ ^14^ ^]^ These spontaneous changes in tumors to adapt to physical sensitization will weaken the radiotherapy efficacy and ultimately enhance the tolerance and resistance of radiotherapy.^[^ ^15^ ^]^ Therefore, the development of radiosensitizers with both physical and biochemical potentiation is expected to break through the bottleneck of radiotherapy.

The complicated microenvironment of tumor tissues is an important reason for limiting radiotherapy to achieve high therapeutic efficacy.^[^ ^5^, ^16^ ^]^ Among them, the redox homeostasis of the TME, as a “stabilizer”, plays an important role in maintaining the physiological processes of tumor cell growth, metabolism, and proliferation.^[^ ^17^ ^]^ When oxidative species, such as reactive oxygen species (ROS), excessively accumulate in cells to disrupt the redox homeostasis, which in turn leads to oxidative damage of lipids and DNA, and ultimately induces oxidative stress and tumor cell death.^[^ ^18^ ^]^ Radiotherapy, as one of the important exogenous modalities for inducing the production of oxidative species in tumor cells, triggers the radioactive decomposition of water molecules to produce large amounts of ROS.^[^ ^19^ ^]^ In this process, tumor cells will demonstrate enhanced reductive substance production capacity to maintain redox homeostasis in the TME and protect tumor cells from oxidative stress.^[^ ^20^ ^]^ Among them, glutathione (GSH) is one of the major reducing substances that eliminate oxidative stress in tumor cells,^[^ ^21^ ^]^ and it greatly limits ROS‐mediated sensitivity to radiotherapy.^[^ ^22^ ^]^ For example, Zhao et al. utilized the pro‐ROS and GSH‐depleting properties of the PVP‐Bi_2_Se_3_@Sec nanosystem to disrupt the redox balance of TME and achieve sensitization to radiotherapy.^[^ ^23^ ^]^ Therefore, it is crucial to restore the biochemical sensitivity of tumor cells in radiotherapy by disrupting the tumor redox homeostasis.

**Scheme 1:**
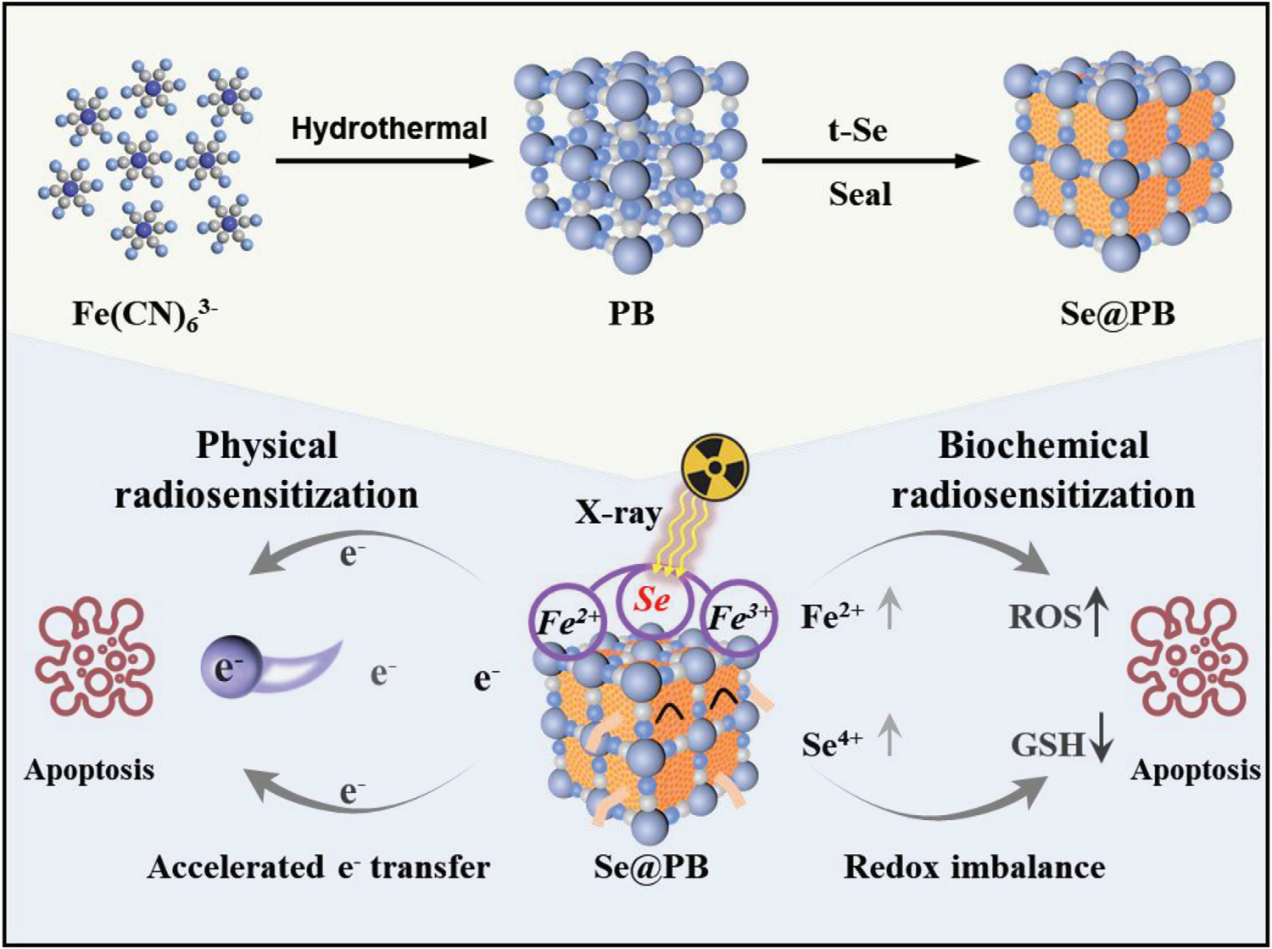
X‐ray‐response properties of Se@PB nano‐heterojunction containing Fe^2+^‐Se‐Fe^3+^ chain exert physical and biochemical radiosensitization by accelerating electron transfer and redox dyshomeostasis.

Development of radiosensitizers with high energy deposition efficiency, electron transfer, and redox perturbation will facilitate the simultaneously achieving physical and biochemical potentiation in tumor radiotherapy.^[^ ^24^ ^]^ For instance, Zhang et al. found that the Fe^2+^‐O‐Fe^3+^ electron chain plays an important role in transferring electrons to Fe_3_O_4_ nanozymes surface and enabling a sustained peroxidase‐like catalytic reaction.^[^ ^25^ ^]^ Selenium (Se), as chemical element of oxygen group, has a loose electronic structure that contributes to the generation of high‐energy electrons and secondary photons under X‐ray radiation. Furthermore, the semiconducting properties of Se that are favorable for the formation of nanoheterojunctions, which gives an advantage in accelerating electron transfer to enhance radiotherapy.^[^ ^26^ ^]^ Prussian blue (PB), as a promising organometallic coordination polymer in biomedical field, its face‐centered cubic unit cells are composed of ferrous (Fe^2+^), ferric (Fe^3+^), and cyanide ions, and the space group is generally referred as Fm3m.^[^ ^27^ ^]^ Herein, we doped Se into PB nanoparticles with mixed valence state (Fe^2+^/Fe^3+^), which developed heterojunction nanosystems (Se@PB) containing Fe^2+^‐Se‐Fe^3+^ electron transfer chains. Among them, the construction of Fe^2+^‐Se‐Fe^3+^ electron transfer chain can enhance the photocurrent effects under X‐ray radiation and promote the transfer of high‐energy electrons, which solely focus to improve the physical sensitivity of radiotherapy. Meanwhile, Se introduction increased the Fe^2+^/Fe^3+^ ratio and promote the conversion of Se to a high valence state (+4), which is beneficial to consume the intracellular reducing substances (such as GSH) and disturb the redox balance of the TME, then in turn exclusively focus on the biochemical sensitivity of radiotherapy. Ultimately, the Se@PB nano‐heterojunction acts as a superior radiosensitizer to drive ROS over production in tumor cells, further induce mitochondrial dysfunction and DNA damage, thus enhance X‐ray induced cell apoptosis and synergistic cervical cancer radiotherapy (**Scheme** **1**).

## 2 Results and Discussion

### 2.1 Synthesis and Characterization of Se@PB Nano‐Heterojunction

**Figure 1:**
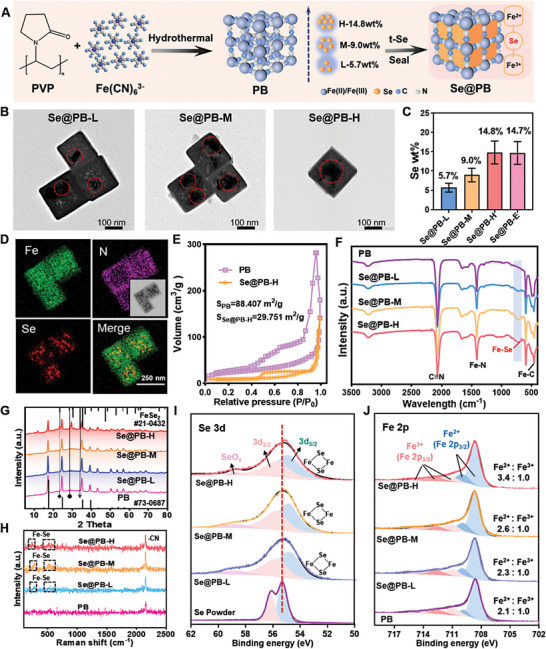
Fabrication process of Se@PB nano‐heterojunction and characterizations of Fe^2+^‐Se‐Fe^3+^ chain. A) Schematic illustration of synthetic process of Se@PB nanoplatform. B) TEM images of Se@PB nanoplatform with different Se ratios (L, M, and H represent low, middle, and high Se content respectively). Insert data are Se percentage content. C) Percentage of Se weight in different nanosystems (Se@PB‐S represent the equilibrium state of Se). D) Element mapping of Se@PB‐H. E) Isothermal adsorption–desorption curves of PB and Se@PB‐H. F) FT‐IR, G) XRD, and H) Raman spectra of Se@PB nanoplatform with different Se content. XPS spectra of I) Se 3d and J) Fe 2p.

Based on the reducibility as well as the mobility of Se powder (t‐Se) in the molten state, we obtained Se@PB nanoplatforms by doping Se into PB nanoparticles with mixed valence state (Fe^2+^/Fe^3+^) in a vacuum‐sealed environment (**Figure** **1**A). And the Se@PB nanosystem with a series of Se doping ratios were controllably synthesized by gradually increasing the feeding mass (w/w) of Se and PB nanoparticles, and were named Se@PB‐L, Se@PB‐M, and Se@PB‐H, respectively (where L, M, H represent low, medium, and high Se content, respectively). We then characterized the morphology and chemical structure of these Se@PB nanosystem by different microscopic and spectroscopic analyses. As shown in the results of transmission electron microscopy (TEM), the three Se@PB nanoparticles maintain the same morphology of PB after different ratios of Se doping, and appear some Se nanoclusters (indicated by red circles) inside PB nanoparticles, suggesting that the Se was successfully combined into the frame structure of PB nanoparticles (Figure 1B). The results of inductively coupled plasma mass spectrometry (ICP‐MS) showed that the Se mass percentages were ≈5.7%, 9.0%, 14.8%, and 14.7% for Se@PB‐L/M/H/E, respectively, suggesting that the Se content in PB at Se@PB‐H reaches saturation and does not continue to rise with increasing Se inputs (Figure 1C). After that, to illustrate the spatial relationship between Se and PB nanoparticles, we performed an elemental mapping analysis for Se@PB‐H. As shown in Figure 1D, the signals of Se elements in the Se‐cluster particles of the bright field (inset image) overlap with some Fe elements, indicating that Se may partially interact with Fe in the PB framework. The results of isothermal adsorption–desorption curves showed that the specific surface area and pore volume decreased after Se doping, suggesting that Se successfully entered and doped into the structure of PB nanoparticles. Among them, the increase of pore diameter in the nanoparticles after Se doping may be due to the small pores of PB nanoparticles being occupied by Se atoms, which lead to an increase in the proportion of residual macropores in the nanosystem (Figure 1E; Table S1, Supporting Information). Subsequently, we further explored the changes in PB structure after Se doping by spectroscopic characterization techniques. First, the ultraviolet–visible spectrum (UV–vis) revealed the absorption no difference after Se doping, suggesting the stability of the PB framework (Figure S1, Supporting Information). Similarly, the Fourier transform infrared spectroscopy (FT‐IR) results showed that the positions of the characteristic peaks of PB, including Fe‐N (1409 cm^−1^), Fe‐C (465 cm^−1^), and ‐CN (2069 cm^−1^), were not significantly shifted after Se doping. Differently, the special peak of Fe‐Se appeared at 733 cm^−1^ in the spectra of Se@PB, and their intensities were enhanced with the increase of Se doping content (Figure 1F). In addition, the diffraction peaks of Fe‐Se were gradually revealed in X‐ray diffraction (XRD) spectra of Se@PB by comparing the standard patterns of FeSe_2_, especially in the high content of Se doping of Se@PB‐H (Figure 1G). Furtherly, the characteristic peaks at 257, 518, and 585 cm^−1^ in the Raman spectra of Se@PB can be attributed to the Fe‐Se stretching vibration (Figure 1H). These characterization data suggested that the heterojunction between Se and PB was formed. Moreover, these spectroscopic data also confirm the existence of the Fe–Se structure in Se@PB, and the mixed Fe^2+^/Fe^3+^ valence state in PB provides the basis for the Fe^2+^‐Se‐Fe^3+^ chain. For further confirmation, we performed Se and Fe binding energy analysis using X‐ray photoelectron spectroscopy (XPS). As shown in Figure 1I, compared with Se powder, a new characteristic peak at 59.3 eV appears on the Se 3d spectrum of Se@PB, which is attributed to the high valence of Se (SeO_x_). Meanwhile, the binding energy of the main peaks of Se@PB‐L, M, and H are reduced, and the Fe^2+^/Fe^3+^ ratio was increased to 3.4:1 from 2.1:1 after Se doping (Figure 1J), which suggests that Se can capture electrons from Fe^2+^ and provide electrons to Fe^3+^ to form a Fe^2+^‐Se‐Fe^3+^ electron transfer chain in the Se@PB structure. Overall, the successfully synthesized Se@PB nano‐heterojunction with a Fe^2+^‐Se‐Fe^3+^ electron transfer chain will provide a prerequisite for X‐ray energy transfer and electron transport in radiotherapy.

### 2.2 Se@PB Nano‐Heterojunction Enhances Radiotherapy Physical Sensitivity

**Figure 2:**
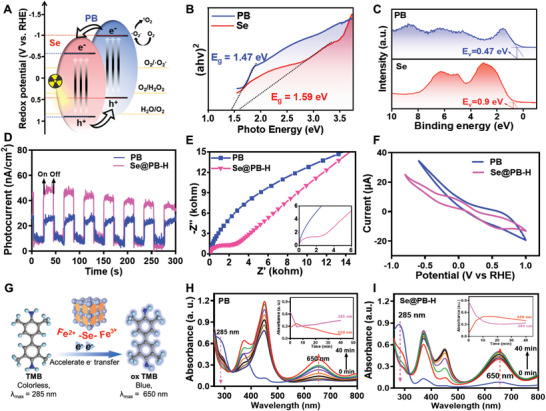
Heterogeneous structure containing Fe^2+^‐Se‐Fe^3+^ chain enhances radiotherapy physical sensitivity. A) Schematic diagram of Se@PB nano‐heterojunction enhancing separation and migration of electron−hole pairs under X‐ray. B) Bandgaps of PB NPs and Se obtained by UV−vis DRS and the Kubelka−Munk formula. C) Valence‐band spectrum of PB and Se. D) Photocurrent of PB and Se@PB‐H under full‐spectrum light irradiation. E) Impedance spectra and F) cyclic voltammetry curve of PB and Se@PB‐H. G) Schematic diagram of the Fe^2+^‐Se‐Fe^3+^ chain‐promoted electron transfer in Se@PB as detected by TMB reaction kinetics. H,I) TMB as a probe to investigate the chemical dynamics of electron transfer processes of PB and Se@PB‐H.

As a typical semiconductor element, Se is prone to form a heterogeneous structure when binding with PB nanoparticles, which effectively adjusts the electronic structure through the synergistic effect of Fe^2+^‐Se‐Fe^3+^ chains, promotes the separation efficiency of electron‐hole pairs under X‐ray radiation, and enhances the physical sensitivity of radiotherapy (**Figure** **2**A). To investigate the structural properties of Se@PB nano‐heterojunction, we have analyzed the energy bandgaps of Se and PB by solid‐state ultraviolet‐visible diffuse reflectance spectroscopy (UV–vis DRS). As shown in Figure 2B, the energy bandgaps of Se and PB are 1.57 and 1.47 eV, respectively, and the forbidden bandgap width of the Se@PB‐H nanosystem was further reduced to 1.09 eV (Figure S2, Supporting Information), which might be attributed to the lowering of the electron‐leaping energy barrier due to the formation of nano‐heterostructure between Se and PB. In addition, we analyzed the valence band spectra to obtain the valence band (VB) energies of 0.9 eV and 0.47 eV for Se and PB, respectively (Figure 2C), and further calculated the conduction band energies of Se and PB to be −0.69 and −1.0 eV, respectively, which are consistent with the characteristics of type II heterojunctions. This heterostructure of Se@PB‐H exhibits a stronger photocurrent response than that of PB nanoparticles (Figure 2D), which suggests that Se doping enhances the separation efficiency of electron‐hole pairs at the nanomaterial surface. Importantly, we evaluated the transport impedance of e^−^ and h^+^ at the Se@PB heterojunction interface by electrochemical impedance. As shown in Figure 2E and Figure S3 (Supporting Information), the arc diameter gradually decreases with increasing Se doping content, suggesting a decrease in the interfacial charge transfer resistance. Meanwhile, we compared the redox properties of PB and Se@PB‐H by cyclic voltammetry curves. As shown in Figure 2F, compared to the only presence of oxidation peaks in PB spectra, the redox peaks of Se@PB‐H nanosystems suggest that they can exert more durable biological effects in vivo as a reversible reaction system. Therefore, Se plays an important role in accelerating electron transfer to regulate redox reactions by modulating the Se@PB heterostructure.

Subsequently, to assess the role of the heterogeneous structure in the electron transfer, we further monitored the chemical kinetic process of electron transfer by using 3,3′,5,5′‐tetramethylbenzidine (TMB) as a probe (Figure 2G). In this case, the oxidation of colorless TMB to blue oxTMB belongs to the process of electron loss, and thus the electron transfer speed on the surface of nanomaterial can be reflected by the enhancement of the intensity of oxTMB (650 nm) or the weakening of TMB (285 nm) as measured by UV spectroscopy. As shown in Figure H‐I, Se@PB‐H promoted a higher level of oxTMB generation than that of PB nanoparticles, and Se@PB‐H induced a longer‐lasting oxTMB accumulation (inset data), which was mainly attributed to the fact that the Se@PB heterostructure effectively inhibits the electron and hole complexation. Meanwhile, the catalytic process using hydrogen peroxide (H_2_O_2_) as substrate also demonstrated that Se@PB‐H has a longer‐lasting and higher substrate reactivity than PB, which is attributed to the high electron transfer of Se@PB‐H containing Fe^2+^‐Se‐Fe^3+^ chain (Figure S4, Supporting Information). The enhanced surface electron migration properties of Se@PB nano‐heterojunction predicted that they possessed greater advantages than PB in responding to X‐ray and accelerating the participation of electrons in biochemical reactions in vivo.

### 2.3 ROS Overproduction and GSH Depletion to Enhance Biochemical Radiosensitivity

**Figure 3:**
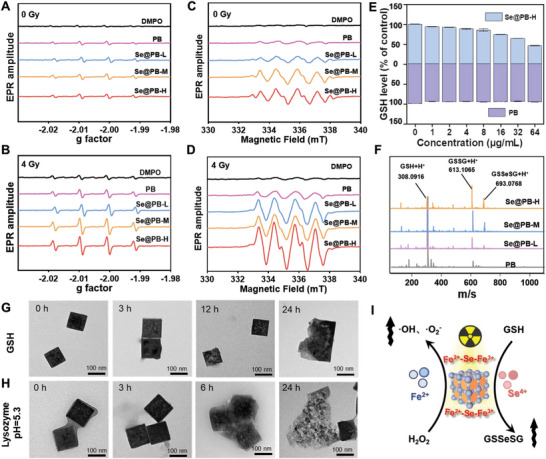
ROS production and GSH depletion to enhance radiotherapy biochemical sensitivity. Level of·OH after PB and Se@PB nanosystem (80 µg mL^−1^) treated with A) 0 Gy B) 4 Gy using DMPO as trapping agent. Levels of ·O_2_^−^ production after PB and Se@PB nanosystem (80 µg mL^−1^) treated with C) 0 Gy D) 4 Gy using DMPO as trapping agent. E) The ability of PB and Se@PB‐H to deplete GSH at different concentration. F) High‐resolution mass spectra after Se@PB nanosystem reacting with GSH. G) TEM images of Se@PB‐H nanosystem (200 µg mL^−1^) after incubation with GSH (2 mM) for different time. H) TEM images of Se@PB‐H nanosystem (200 µg mL^−1^) after incubation with lysozyme (2 mg mL^−1^) at pH 5.3 for different time. I) Schematic demonstration of the Fe^2+^‐Se‐Fe^3+^ chain driving ROS overproduction and GSH depletion under X‐ray to enhance biochemical sensitivity to radiotherapy.

Tumor cells maintain the redox homeostasis of the intracellular environment by regulating ROS and GSH, thereby greatly limiting the ROS‐mediated radiotherapy effect and reducing the biochemical sensitivity of radiotherapy.^[^ ^28^ ^]^ While the Fe^2+^‐Se‐Fe^3+^ chain in Se@PB nano‐heterojunction will play an important role in accelerating the electron transfer between the nanomaterials and the substrates (such as H_2_O_2_ and GSH), and endow the Se@PB nanosystem with excellent redox reaction performance. Therefore, we then explored the performance of Se@PB nanosystem in promoting ROS overproduction and GSH depletion in vitro. As shown in **Figure** **3**A,B, we tracked the hydroxyl radical (·OH) signal captured by DMPO (4,4,5,5‐dimethyl‐1‐pyrroline‐N‐oxide) by electron spin resonance (ESR), and found that the Se@PB nanosystem significantly increased the intensity of DMPO‐OH signal peaks. Importantly, the level of ·OH production was further increased under X‐ray radiation at 4 Gy, especially Se@PB‐H showed the most significant synergistic enhancement, which achieves a threefold higher ·OH level (Figure S5, Supporting Information). At the same time, we observed similar results for superoxide anion (·O_2_^−^) detection, which showed a much higher generation of ·O_2_^−^ induced by Se@PB nanosystem with X‐ray radiation (Figure 3C,D; Figure S6, Supporting Information). Therefore, Se@PB nano‐heterogeneous, especially Se@PB‐H NPs, can efficiently induce a large accumulation of ROS through pro‐Fenton reaction due to the increase of Fe^2+^/Fe^3+^ ratio, and exhibit a synergistic benefit combined with X‐ray. Notably, in response to massive ROS accumulation, tumor cells strengthen the antioxidant defense system (such as GSH) to maximize the elimination of ROS‐induced functional damage or cell death. Therefore, based on the oxidation induced by the high valence Se in Se@PB nano‐heterojunction, we further investigate the GSH depletion to evaluate the ability to disrupt the redox homeostasis by Se@PB. As shown in Figure 3E, we observed that PB nanoparticles had almost no ability to deplete GSH, whereas Se@PB‐H exhibited concentration‐dependent enhanced GSH depletion ability. In addition, to reveal the action mechanism of Se in the depletion of GSH, we analyzed the products after co‐treatment of Se@PB nanosystem and GSH by high‐resolution mass spectrometry. As shown in Figure 3F, we observed the enhanced oxidizing GSH (GSSG) signaling and decreased GSH signaling in the reaction products of Se@PB nanosystem with increasing Se doping content, whereas PB promoted only weak GSSG production. More importantly, we also recognized a new signal peak of GSSeSG in the reaction products, and the signal intensity enhanced with the increase of Se content in Se@PB nanosystem. Thus, the realization of GSH depletion by Se@PB nano‐heterojunction can be attributed to the GSH oxidation to GSSG and GSSeSG by the high valence Se, instead of PB. The TEM results revealed the response behavior of Se@PB‐H nanoparticles in a GSH (2 mm) environment. As shown in Figure 3G, the Se@PB‐H framework did not undergo significant disintegration at 12 h, whereas an obvious disruption began to appear at 24 h, indicating the slower GSH response behavior of Se@PB‐H, which contributes to the long‐lasting catalytic effect of Se@PB‐H in tumor cells. Furtherly, Se@PB‐H showed a stepwise responsive behavior in lysozyme and weakly acidic simulated physiological environments, which also suggested the good bioresponsiveness of Se@PB‐H nanosystems within cells or tumor tissues (Figure 3H). Overall, the increase of Fe^2+^/Fe^3+^ ratio and conversion of Se to a high valence state in Se@PB nano‐heterojunction are conducive to realizing the pro‐Fenton reaction and GSH oxidation reaction, thereby triggering the ROS overproduction and disturbing the redox balance of the TME to enhance the biochemical sensitivity of radiotherapy (Figure 3I).

### 2.4 In Vitro Evaluation of Radiotherapy Sensitization of Se@PB (R) Nanoheterojunctions

**Figure 4:**
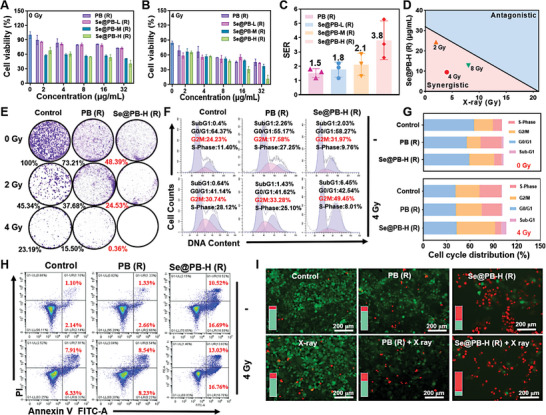
In vitro evaluation of radiotherapy sensitization of Se@PB (R) nanoheterojunctions. PB (R) and Se@PB (R) nanosystem‐induced cytotoxicity in HeLa cells at 72 h. A) without X‐ray radiation B) with X‐ray radiation of 4 Gy. C) SER of PB (R), Se@PB‐L (R), Se@PB‐M (R) or Se@PB‐H (R) under X‐ray (4 Gy). D) An isobologram analysis of Se@PB‐H (R) in cell activity at different X‐ray doses. E) Photographs of colony formation after treatments of PB (R) or Se@PB‐H (R) (1 µg mL^−1^) combination with/without X‐ray. F) The effects of PB (R) and Se@PB‐H (R) (8 µg mL^−1^) under X‐ray irradiation (4 Gy) on the cell cycle of HeLa tumor cells treated for 48 h. G) Quantitation analysis of cell cycle distribution. H) Cell apoptosis induced by Se@PB (R) nanosystem (8 µg mL^−1^) combined with/without X‐ray for 48 h. I) Images and the inner histogram are green/red fluorescence ratios of HeLa cells after the different treatments (green and red represent live cells and dead cells).

To enhance the biocompatibility and tumor targeting of Se@PB nano‐heterojunction, we further used phospholipid polyethylene glycol (DSPE‐mPEG) covalently coupled with RGD peptides to modify the surface of Se@PB. First, we evaluated the biocompatibility of Se@PB‐H (R) by hemolysis experiments, and found that Se@PB‐H (R) exhibited good hemocompatibility, and induced only less than 10% hemolysis even at a concentration of 100 µg mL^−1^ (Figure S7, Supporting Information). Furthermore, the results of in vivo imaging tracking analysis of indocyanine green (ICG) labeled Se@PB‐H (R) in HeLa tumor‐bearing mice also showed that RGD modification significantly increased the enrichment of nanoparticles at the tumor site (Figure S8, Supporting Information). As shown in Figure S9 (Supporting Information), the targeted‐modified Se@PB‐H (R) also demonstrated the time‐dependent accumulation of Se content within the tumor cells, and the Se content at 12 h was approximately twofold higher than that of the non‐targeted group. About anti‐tumor activity study, Se@PB‐M (R), and Se@PB‐H (R) exhibited much higher anticancer activity than that of the free PB nanoparticles and the lower Se‐loading of Se@PB‐L (R), suggesting the Se content‐dependent cancer‐killing effects of Se@PB nano‐heterojunction (**Figure** **4**A). Importantly, the HeLa cell viability was further decreased after being treated by Se@PB‐H (R) combined with X‐ray (Figure 4B). Among them, the IC_50_ of Se@PB‐H (R) combined with 4 Gy decreased to 11.3±2.4 µg mL^−1^, significantly different from 36.9±4.9 µg mL^−1^ at 0 Gy. Further, the sensitivity enhancement ratio (SER) of these nano‐heterojunctions was calculated, and found that the SER values show a gradual upward trend with increasing Se doping content, reaching ≈1.76, 2.11, 3.81 for Se@PB‐L/M/H (R), respectively. (Figure 4C). Notably, compared with other Se‐containing nanosystems (such as MoSe_2_ NF‐RGD, SeAuFe‐EpC, and Bi_2_Se_3_, etc.), the Se@PB (R) nanosystem not only exhibits higher SER values, but also possesses a high safe index, which fully confirms the efficiency and safety of the Se@PB (R) nanosystem (Table S2, Supporting Information). Meanwhile, the results of the equivalent analysis showed that Se@PB‐H (R) exhibited a much higher synergistic effect combined with X‐ray at different irradiation doses (2, 4, 8, 10 Gy) (Figure 4D). Furthermore, we also evaluated the radiosensitization effects of Se powder as a comparison, and found that it did not show significant tumor growth inhibition combined with or without X‐ray radiation (Figure S10, Supporting Information). These results suggest that the heterogeneous structure with Fe^2+^‐Se‐Fe^3+^ chain formed by Se doping is the main reason for the significantly enhanced antitumor activity and radiosensitization ability of Se@PB (R) nano‐heterojunction.

In addition, we also explored the selective tumor killing of Se@PB (R) nano‐heterojunction by detecting its cytotoxicity on normal cervical cell lines (ECT). As shown in Figure S11 (Supporting Information), these Se@PB (R) nanosystem with different Se contents showed low cytotoxicity against ECT cells even combined with X‐ray treatment, which indicated that Se@PB (R) nanosystems have great selective killing for tumor cells as well as high safety for normal cells. Further, we also evaluated the radiotherapy sensitizing ability of Se@PB (R) nanoplatform by cell cloning experiments, and found that the Se@PB‐H (R) nano‐heterojunction exhibited stronger inhibition for cell colony formation than that of PB nanoparticles, and further enhanced after combined with X‐ray irradiation (Figure 4E). We then also observed a significant increase in the proportion of HeLa cells blocked in the G2/M phase (which is more sensitive to X‐rays) after treated with Se@PB‐H (R) and radiotherapy (Figure 4F,G), showing a Se‐dependent rising tendency (Figure S12, Supporting Information). Therefore, the G2/M phase effect induced by the Se@PB‐H (R) nanoplatform in combination with radiotherapy is amplified, thereby inhibiting tumor cell proliferation. In addition, the results of Annexin V‐FITC/PI double staining assay showed that radiotherapy alone caused only ≈14.24% apoptosis, whereas Se@PB‐H (R) combination radiotherapy achieved up to 29.79% apoptosis, which was significantly higher than the combined therapeutic effect of other Se@PB nanosystem (Figure 4H; Figure S13, Supporting Information). Furthermore, calcein AM and propidium iodide (PI) double staining assay also visually demonstrated that Se@PB‐H (R) combined with X‐ray radiation significantly induced cell death than that treated by PB (R) nanoparticles, which was reflected by the increase of the red fluorescence intensity (Figure 4I). Therefore, these results fully demonstrated that Se doping could effectively enhance Se@PB (R) nano‐heterojunction to induce HeLa cells growth inhibition, cell cycle blockade, and apoptosis, thereby ultimately realizing the radiosensitization effects.

### 2.5 Fe^2+^ Accumulation Triggers ROS Production and Mitochondrial Damage

**Figure 5:**
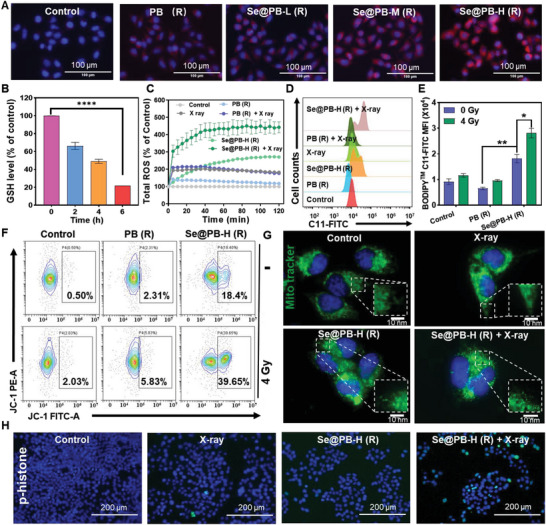
Fe^2+^ accumulation drives ROS production and mitochondrial damage. A) Images of HeLa cells intracellular Fe^2+^ levels detected by FerroOrange (1 µm) treated with PB (R) and Se@PB (R) nanosystem (8 µg mL^−1^) for 4 h. B) Intracellular GSH level after treatment with Se@PB‐H (R) (8 µg mL^−1^) for 0, 2, 4, 6 h. C) ROS generation in HeLa cells after exposure to Se@PB‐H (R) (8 µg mL^−1^) and in combination with radiotherapy (4 Gy). D) Lipid peroxidation levels of HeLa cells under different treatment conditions. E) Quantification of lipid peroxidation levels. Data represented as mean ± SD (*n* = 3). Significant difference was calculated by one‐way ANOVA (^*^ *P* < 0.05, ^**^ *P* < 0.01). The mitochondrial F) membrane potential and G) images of HeLa cells incubated with different groups. H) Level of p‐histone after treating with different groups (PB (R), Se@PB‐H (R): 8 µg mL^−1^, X‐ray: 4 Gy).

Based on the Se@PB (R) heterostructure in triggering ROS overproduction and GSH depletion, we then further explored their radiosensitization mechanism in tumor cells. The pro‐Fenton reaction of Fe^2+^ provides favorable conditions for the ROS overproduction, thus we detected the changes in intracellular Fe^2+^ level by the probe of FerroOrange. We found that Se@PB (R) treatment resulted in more pronounced Fe^2+^ accumulation in tumor cells, especially Se@PB‐H (R) (**Figure** **5**A). On the other hand, we also detected the expression level of intracellular GSH, and found that Se@PB‐H significantly induced the time‐dependent reduction of GSH (Figure 5B). Furtherly, the increase of Fe^2+^ and reduce of GSH together accelerate the ROS accumulation. Therefore, we monitored the changes of intracellular ROS levels induced by PB (R) and Se@PB (R) nanoplatforms, and found that Se@PB (R) can significantly induce ROS overproduction and exhibit Se content‐dependent elevation (Figure 5C; Figure S14, Supporting Information). Importantly, Se@PB (R)‐induced ROS production was further exacerbated under X‐ray radiation, which produced about 4.4‐fold higher than that of the control group within 2 h (Figure S15, Supporting Information). These excessive ROS will further react with macromolecules such as cellular phospholipids, enzymes, and membrane receptor‐associated unsaturated fatty acid side chains and nucleic acids to cause lipid peroxidation, which in turn disrupts the normal physiological functions of the cell. Therefore, we then analyzed the intracellular lipid peroxidation levels induced by Se@PB nano‐heterojunction combined with X‐ray by flow cytometry. As shown in Figure 5D,E, the lipid peroxidation level caused by Se@PB‐H (R) was significantly higher than that of the PB (R) group without Se‐doping, and further increased under X‐ray radiation. Furtherly, the overproduction of ROS also leads to a decrease in mitochondrial membrane potential (ΔΨm), inducing mitochondrial dysfunction. Therefore, we detected the intracellular ΔΨm and found that X‐ray co‐treatment group with Se@PB‐H (R) induced ≈39.65% change in ΔΨm, which was superior to the co‐treatment group with PB (5.83%) (Figure 5F). More intuitively, the decrease in mitochondrial potential was observed after being co‐treated by Se@PB‐H (R) and X‐ray, which reflected as an increase in green fluorescence intensity and a decrease in red fluorescence intensity (Figure S16, Supporting Information). Meanwhile, the mitochondrial network was fragmented into the point structure after being treated by Se@PB‐H (R) combined with X‐ray radiation (Figure 5G). Furthermore, this radiosensitization therapy significantly up‐regulated the expression of p‐histone, a special mark of DNA damage (Figure 5H), which effectively induced cell apoptosis and enhanced the tumor radiotherapy. In summary, these results indicated that Se@PB‐H (R) nano‐heterojunction containing Fe^2+^‐Se‐Fe^3+^ chain triggered electron transfer under X‐ray radiation to induce ROS overproduction and enhance radiotherapy physical sensitivity, then further induced GSH depletion and Fe^2+^ accumulation in tumor cells to disturb the redox balance and enhance biochemical sensitivity of radiotherapy.

### 2.6 Antitumor Activity of Se@PB‐H (R) Combined with X‐Ray In Vivo

**Figure 6:**
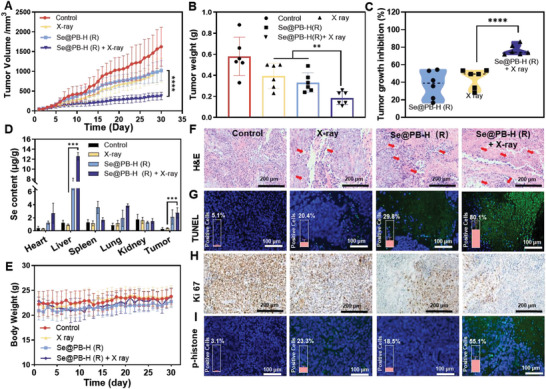
Antitumor activity of Se@PB‐H (R) combined with X‐ray in vivo. A) Tumor growth curves in different groups (Se@PB‐H (R): 5 mg kg^−1^, X‐ray: 2 Gy, every other day). Data are represented as mean ± SD (****: *P* < 0.0001, *n* = 6 mice per group). B) Tumor weight and C) tumor growth inhibition ratio at 30 days in different treatment groups (**: *P* < 0.01, *n* = 6). D) Se content distribution in different treatment groups after 30 days (***: *P* < 0.001, n = 3). E) Weight change curves in different groups within 30 days (n = 6 mice per group). F) Histopathological features of tumors in different groups (H&E staining). G) TUNEL assay was applied to assess apoptosis in different groups of tumors. H) Immunohistochemical analysis of Ki‐67 in different treatment groups. I) Immunofluorescence images and quantification analysis of p‐histone.

Based on the physical and biochemical sensitizing properties of Se@PB (R) nano‐heterojunction for radiotherapy, we further comprehensively evaluated its sensitization effects and mechanism in HeLa tumor‐bearing mice. As shown in **Figure** **6**A and Figure S17 (Supporting Information), compared to the saline group, both the Se@PB‐H (R) and X‐ray groups treated alone inhibited tumor growth to some extent, whereas the Se@PB‐H (R) combined with the radiotherapy group showed the most significant tumor inhibition, which showed that the tumor volume was significantly smaller than in the other treatment group alone. In addition, the tumor weight of the Se@PB‐H (R) combined with the X‐ray treatment group was significantly lower than the other treatment group alone (Figure 6B), and the tumor inhibition reached ≈76.7% (Figure 6C). Subsequently, we also measured the Se content in various organs by ICP‐MS to evaluate the distribution and accumulation of the nanodrug in the treated mice. As shown in Figure 6D, compared with the saline group or X‐ray group, there was a significant Se enrichment in tumor sites after injection with Se@PB‐H (R), which may be due to the cancer targeting modification on the surface of nano‐heterojunction to promote drug uptake. Furthermore, there was no significant change in body weight among the treated mice (Figure 6E), suggesting the high safety of Se@PB‐H (R) as a radiosensitizer in the combination therapy. To further explore the action mechanism of Se@PB‐H (R) radiosensitization therapy, we performed pathological and immunohistochemical analyses of tumor tissues from each treatment group.

As shown in the results of hematoxylin‐eosin (H&E), the Se@PB‐H (R) combined with radiotherapy significantly increased the necrosis areas (indicated by red arrows) in the tumor tissues (Figure 6F), and further induced the most significant tumor cell apoptosis, which reflects as the green fluorescence increase of TUNEL (Figure 6G). We then also detected the expression of Ki‐67, an indicator of tumor cell proliferation, and found that the malignant proliferation of tumor cells was significantly reduced after Se@PB‐H (R) combined with X‐ray treatment, which was closely related to its tumor growth inhibition (Figure 6H). Importantly, to verify that Se@PB‐H (R) as a radiotherapy sensitizer synergistically enhanced the DNA damaging effect, we performed an immunofluorescence analysis of p‐histone. As expected, the combined treatment group of Se@PB‐H and X‐ray demonstrated the most significant DNA damage effect with the expression increase of p‐histone (Figure 6I). Overall, these results indicated that Se@PB‐H (R) can act as an excellent radiosensitizer to promote X‐ray‐induced DNA damage and then enhance tumor necrosis and apoptosis in vivo.

## 3 Conclusion

Clinically, tumor radiotherapy, still faces bottlenecks of radiation tolerance, immunosuppression, and poor prognosis, thus the demand for safe and efficient radiosensitizers is very urgent.^[^ ^13c^ ^]^ Nano‐heterojunction, not only influences the energy deposition efficiency of radiotherapy through accelerating the electron transfer, but also interferes with the biochemical reaction processes of TME, together promoting radiosensitivity of the tumor.^[^ ^13b^ ^]^ However, in which way the nano‐heterojunction achieves electron transfer and through which substance interferes with the TME to exert its anti‐tumor mechanism has not been intensively investigated yet. In this work, we reported nano‐radiosensitizers with strong electron transfer and redox perturbation to simultaneously achieve physical and biochemical potentiation in tumor radiotherapy by enhancing secondary electron transport under X‐ray radiation through the electron transfer chain. Our work is summarized as follows: a) First, we have constructed a Se@PB nano‐heterojunction with Fe^2+^‐Se‐Fe^3+^ electron‐transporting chain and chemical valence state intra‐modulation properties by doping Se into PB nanoparticles with mixed valence state (Fe^2+^/Fe^3+^). b) The Fe^2+^‐Se‐Fe^3+^ electron transfer chains performed excellent performance in enhancing photocurrent effects under X‐ray radiation and reducing the resistance of electron transport, and finally improved the physical sensitivity of radiotherapy. c) The increased Fe^2+^ and Se^4+^ ratio enhanced ROS accumulation under Fenton reaction and the intracellular GSH level depletion, thereby disturbing the redox balance of the TME, then in turn restored the biochemical sensitivity of radiotherapy. d) The Se@PB nano‐heterojunction acts as a superior radiosensitizer and demonstrated a high SER value (3.8) and SI (19.4) by causing cell cycle arrest and apoptosis in cervical cancer cells. e) Se@PB nano‐heterojunction drove ROS overproduction based on chemodynamic process of Fe^2+^ in tumor cells, these ROS further induced lipid peroxidation, mitochondrial dysfunction, and DNA damage, thus enhancing X‐ray induced cell apoptosis and synergistic cervical cancer radiotherapy. f) Finally, Se@PB nano‐heterojunction simultaneously achieved physical and biochemical potentiation in tumor radiotherapy in vivo by accelerating electron transfer and inducing redox perturbation, and demonstrated superior therapeutic efficacy by inducing cell apoptosis, inhibiting proliferation, and enhancing DNA damage in tumor tissue cells. Overall, this work will provide new ideas and thoughts for the design of nano‐heterojunction based on the enhancement of radiotherapy physical and biochemical sensitivity through heterojunctions containing electron transport chains and redox balance disturbances, as well as scientific references for elucidating the electron transport mechanisms of radiosensitizers.

In the future, we will continue to explore the exciting and unknown possibilities in this work to fill the deficiencies as well as to push forward the design and development of radiosensitizers with potential for clinical translation. The shortcomings and perspectives of this study are as follows: First, the mechanism of nano‐heterojunction action in vitro and in vivo still needs to be elaborated deeply. For example, the monitoring of the process of electron transfer in the Fe^2+^‐Se‐Fe^3+^ electron transport chain needs to be corroborated with time‐precise spectroscopic data. Second, in‐depth in vivo studies are needed. By identifying the key targets of the interaction between nanoheterojunctions and proteins, to explore the relationship between target binding and radiosensitization. In addition, it is necessary to analyze how nanomaterials exert their biochemical sensitization at different levels through histological analyses and validation at the RNA and protein levels. Third, A comprehensive assessment of the biosafety of nanoheterojunctions needs to be performed in detail. Comprehensive studies on toxicology, pharmacokinetics, thermodynamics, and other properties should be carried out to compare the advantages and disadvantages of nanoheterojunctions with those of current clinical drugs, so as to provide more theoretical data to support their entering into the clinical field. Finally, by exploring and decoding the physicochemical and biological properties of the nano‐heterojunction and evaluating its clinical safety, we hope to provide a systematic approach and technical means for the development of sensitizers in radiotherapy. Knowing the shortcomings and advancing, we look to the distant mountains and move forward.

## 4 Experimental Section

### Materials

In this work, and 2′,7′‐dichlorodihydrofluorescein diacetate (DCFH‐DA), JC‐1 fluorescent probe were procured from Sigma Aldrich. Se powder, polyvinylpyrrolidone (PVP), *N*‐hydroxysuccinimide (NHS), 1‐ethyl‐3‐(3‐dimethylaminopropyl) carbodiimide (EDC), ICG, and reduced GSH, 30% H_2_O_2_ and potassium ferricyanide (K_3_[Fe(CN)_6_]) were obtained from Aladdin (Shanghai, China). Moreover, FerroOrange and DMPO were purchased from DOJINDO Laboratories (Shanghai, China). All protein antibodies were purchased from Cell Signaling Technology Co., Ltd. Annexin V‐FITC/PI double staining reagent was from Thermo Fisher Scientific.

### Synthesis of Se@PB Nano‐Heterojunction

PB nanoparticles were prepared in a hydrothermal reaction as described previously.^[^ ^29^ ^]^ First, 3.0 g of PVP and 131.7 mg of K_3_[Fe(CN)_6_ were weighed and dissolved in 20 mL of deionized H_2_O. 20 mL of 0.02 m HCl solution was added to the above system and stirred to form a homogeneous solution. Subsequently, they were transferred to a Teflon‐lined and autoclaved reactor and treated in a muffle furnace at a constant temperature (80°C for 20 h). After cooling to room temperature, the precipitates were collected as products by centrifugation and washed three times with water and ethanol, respectively. Finally, the PB NPs were dried in a vacuum drying oven to obtain PB NPs. To prepare Se@PB nanosystems, 20‐x mg of the above PB NPs were homogeneously mixed with x mg of Se powder (x = 1,2,4), respectively, and were transferred into quartz glass tubes (14 mm in diameter and 280 mm in length). Subsequently, the quartz glass tubes were sealed under a vacuum sealing device and thermostatically treated in a muffle furnace (260°C, keep 1 h). The powder products were collected after natural cooling, and the products at x = 1, 2, 4, 6 were named Se@PB‐L, Se@PB‐M and Se@PB‐H, Se@PB‐E respectively.

### Preparation of Se@PB (R) Nanosystem

Four milligram of RGD cyclic peptide (MW:5 kD) was dissolved in 2 mL of deionized H_2_O, and then EDC (0.2 mg mL^−1^) was added and stirred for 30 min. Subsequently, NHS (0.6 mg mL^−1^) was added and stirred for an additional 30 min to fully activate the carboxyl group on RGD. Twenty milligram of phospholipid polyethylene glycol (DSPE‐mPEG‐NH_2_, MW:2 kD) powder was dispersed in 8 mL of deionized H_2_O and then mixed with the RGD solution and stirred for 24 h. Finally, the solution was collected for dialysis for 48 h (MWCO: 10 kD) to obtain a clarified solution of DSPE‐mPEG‐RGD. Further, 10 mg of the Se@PB NPs was dispersed in 2.5 mL of DSPE‐mPEG‐RGD solution, after stirring for 24 h, the precipitated product was collected by high‐speed centrifugation and washed three times with deionized H_2_O to obtain the targeted peptide‐modified Se@PB nanosystem (Se@PB (R)).

### Characterization of Se@PB Nano‐Heterojunction

Morphology analysis of PB, Se@PB‐L, Se@PB‐M, and Se@PB‐H was carried out under TEM (JEM‐1400 Flash, 120 kV, JEOL Ltd., Japan). Spectroscopic characterization techniques were used to analyze the structural features of PB and Se@PB nanosystems, such as XRD (MiniFlex‐600, Rigaku Corporation, Japan), FT‐IR (PerkinElmer, UATR Two), UV–vis (UV‐2550, Shimadzu Instruments Co. Ltd., Suzhou, China), and XPS (ESCALAB250Xi, Thermo Fisher Scientific Ltd., USA). In addition, ICP‐MS (Agilent 720, USA) was used to quantify Se in Se@PB nanosystems, and a nanoparticle size and zeta potential analyzer (Malvern Instruments Ltd., UK) was used to detect the particle size and potential of Se@PB nanosystems.

### Performance Evaluation of Se@PB Nano‐Heterojunction for Promoting Electron Transfer

To detect the process of accelerated electron transfer in PB and Se@PB nanosystems, a chemical kinetic investigation was performed using TMB as a substrate probe. 20 µL of TMB (5 mm, in DMSO), 10 µL of H_2_O_2_ (30%) were mixed with 2 mL of NaAc‐HAc buffer solution (pH 5.3). PB or Se@PB nanosystems at a concentration of 2 µg mL^−1^ were rapidly added and immediately subjected to kinetic scanning under UV–vis (40 min, 5 min/cycle).

### Determination of ESR

ESR spectrometry was used to detect levels of ·OH and ·O_2_^−^ production with and without X‐ray. PB, Se@PB‐L, Se@PB‐M, and Se@PB‐H NPs at the same concentration (80 µg mL^−1^) were added to an aqueous or methanol H_2_O_2_ solution and reacted in the dark for 10 min. Subsequently, the DMPO trapping agent was added to the above reactions with or without X‐ray (4 Gy) irradiation. Finally, the level of production of each radical was rapidly detected on the ESR.

### Photocurrent Detection

Photocurrent measurements were performed on an electrochemical workstation using a three‐electrode system. In this system, PB and Se@PB nanosystems were used as working electrodes; Hg/HgCl electrode was used as reference electrode; and Pt electrode was used as counter electrode. The electrolyte buffer was PBS solution. Preparation of working electrode: 2 mg of the nanomaterials were mixed and dispersed with 300 µL of anhydrous ethanol, 100 µL of deionized H_2_O, and 10 µL of Nafion by ultrasonication. Subsequently, the mixed liquid was dripped onto a clean conductive glass (ITO, 1 cm × 2 cm) and dried naturally in the room.

### Detection of GSH Depletion In Vitro and In Vivo

Briefly, GSH (100 µm) solutions were incubated with different concentrations of PB or Se@PB‐H NPs, respectively, for 2 h. The remaining GSH was then indicated by the addition of excess 5,5′‐dithiobis (2‐nitrobenzoic acid) (DTNB) solution and the absorbance value at 412 nm was determined. Intracellular GSH levels were assayed after Se@PB‐H (R) incubation with cells for 2,4,6 h. Cells were collected and performed according to the GSH assay kit instructions.

### Cell Culture and Anti‐Tumor Activity Assay

Human cervical cancer cell lines (HeLa, ECT cells) used in the experiments were purchased from American Type Culture Collection (ATCC), USA. They were cultured in DMEM medium containing 10% fetal bovine serum, 100 units/mL penicillin, and 50 units/mL streptomycin. The cell activity assay in this experiment was performed using the MTT assay. HeLa or ECT cells were inoculated in 96‐well plates at a density of 2×104 cells/mL, and co‐treated with PB (R) and Se@PB (R) nanosystems at a concentration gradient after cell attachment. In addition, the cells that needed to be exposed to X‐ray were co‐treated for 6 h, and then applied different doses of radiation. Finally, these cells continued to be cultured for 72 h, and after that, MTT was added for quantitative analysis. Cell viability (%) was calculated as (OD _experimental group_/OD _control group_) × 100%. SER and SI of the different nanomaterials according to the formula: SER = IC_50_ (nanomaterials)/IC_50_ (nanomaterials with RT dose), SI = IC_50_ (normal cells)/IC_50_ (tumor cells)

### Clone Formation Assay

HeLa cells (2000 cells per well) were inoculated into 6‐well plates and adhered for 24 h. Cells were treated with different doses of X‐ray (0, 2, and 4 Gy) in combination with PB (R) and Se@PB‐H (R) (1 µg mL^−1^), and the culture was continued for 14 days. After the formation of cell colonies, the cells were fixed using 4% paraformaldehyde for 20 min at room temperature and stained with crystal violet (0.5 wt.%) for another 20 min. Finally, the cell colonies were photographed through a microscope after natural drying, and the data were quantitatively analyzed using Image J software.

### Assessment of Cellular Uptake of Se@PB and Se@PB (R) Nanosystem

The study assessed the difference in uptake of non‐targeted and targeted nanosystems by HeLa cells using cellular Se content as an assay. HeLa cells were inoculated at a cell density of 1×106 cells/10 cm dish, and they were co‐treated with Se@PB and Se@PB (R) nanosystems respectively for different time (0,2,4,6,8,12 h) after cell attachment. These cells were collected by removing the upper layer of DMEM medium and washing it twice with 5 mL PBS, followed by cell counting. These cells were assayed for Se content using ICP‐MS after treatment with aqua regia.

### Detection of Intracellular Fe^2+^ Level

HeLa cells (2 × 105 cells/mL) were seeded in a 6‐well plate and cultured for 24 h. The next day, PB (R) and Se@PB (R) (8 µg/mL) nanosystem were added and incubated with the cells for 6 h. Remove the medium and add FerroOrange working solution with a concentration of 1 µm, and incubate for 30 min, and these cells can be observed directly under a fluorescence microscope.

### Intracellular ROS Level Assay

HeLa cells (2×105 cells/mL) were inoculated in 96‐well plates and adhered for 24 h. On the next day, different concentrations of PB (R) and Se@PB (R) nano‐systems were added to co‐incubate with the cells for 6 h. Next, DCFH‐DA fluorescent probe was added and co‐treated for 30 min, and the DMEM medium in the wells was removed and 200 µL PBS was added to eliminate the effect of medium. Finally, the changes in ROS levels in the cells within 120 min were recorded using cell multi‐mode reader at excitation and emission wavelengths of 488 and 528 nm. In addition, cells that needed to undergo X‐ray irradiation were co‐incubated for 6 h and then subjected to X‐ray (4 Gy), followed by monitored for ROS production.

### Cell Cycle and Apoptosis Analysis

Flow cytometry was used to detect cell cycle and apoptosis induced by the nanosystem combined with X‐ray. HeLa cells (4×104 cells/mL) in a 10 cm culture dish and wait for the cells to adhere. The next day, 16 µg mL^−1^ of PB (R) and Se@PB (R) nanoparticles were added to co‐treat for 6 h, followed by 4 Gy of X‐ray irradiation and continued culture for 48 h. Finally, the cells were collected and washed twice with pre‐cooled PBS. The cells were double‐stained with Annexin V‐FITC and PI according to the experimental method in the assay kit instructions and tested on the machine. In addition, for cell cycle analysis, the collected cells were resuspended in a pre‐cooled 75% ethanol solution and then placed in a 4°C refrigerator overnight. The next day, the ethanol was removed, stained with PI for 15 min in the dark, and finally analyzed on a flow cytometer.

### In Vivo Antitumor and Radiotherapy Sensitization Study

In this study, 30 female BALB/c‐nude mice (6 weeks old) were purchased from GemPharmatech Co., Ltd. All animal experiments were conducted in accordance with the guidelines of the Animal Ethics Committee of Jinan University (Approval number: IACUC‐20230416‐02). To establish the tumor model, HeLa cells (1×107 cells/mL) were suspended in PBS and injected into the right side of mice (100 µL/mice) by subcutaneous injection. When the average volume of the xenograft tumors reached ≈100 mm^3^, the mice were randomly divided into four groups: saline group, saline + X‐ray group, Se@PB‐H (R) group, and Se@PB‐H (R) + X‐ray group. These mice were treated with intravenous administration (100 µL saline or 5 mg kg^−1^ Se@PB‐H (R)) twice a week or concurrently with X‐ray treatment (2 Gy) 6 h after injection. In addition, the mice were monitored daily for body weight and tumor volume. Tumor volume = 1/2 × length × width × width. After 30 days, the mice were euthanized and stripped of the tumors as well as well as the major organs of the mice (heart, liver, spleen, lungs, and kidneys). They were divided into two parts, one was fixed in 4% paraformaldehyde for further immunohistochemistry or immunofluorescence, and the other part was used to detect Se content in tumor and each organ.

### Statistical Analysis

Quantitative data are expressed as mean ± standard deviation. When comparing two or more groups separately, statistical differences were calculated with GraphPad Prism software (version 8) using an unpaired Student's *t*‐test or one‐way analysis of variance (ANOVA). Statistical differences are expressed in ns and are not significant. *P < 0.05, ** *P* < 0.01, ***P < 0.001, ****P < 0.0001.

## Conflict of Interest

The authors declare no conflict of interest.

## Supporting information

Supporting Information

## Data Availability

The data that support the findings of this study are available from the corresponding author upon reasonable request.
