# Supplementary material for: Selenium‐Doped Nanoheterojunctions for Highly Efficient Cancer Radiosensitization
Source: Adv Sci (Weinh). 2024 Jun 3;11(29):2402039. doi: 10.1002/advs.202402039 (PMC11304322; doi:10.1002/advs.202402039)
Supplement: Supplementary file 1 — Supporting Information [file ADVS-11-2402039-s001.pdf]

ADVANCED SCIENCE
Open Access

Supporting Information

for *Adv. Sci.*, DOI 10.1002/advs.202402039

Selenium-Doped Nanoheterojunctions for Highly Efficient Cancer Radiosensitization

Rui Qiao, Zhongwen Yuan, Meijin Yang, Zhiying Tang, Lizhen He and Tianfeng Chen**

Supporting Information

Selenium-doped nanoheterojunctions for highly efficient cancer radiosensitization

Rui Qiao#, Zhongwen Yuan#, Meijin Yang, Zhiying Tang, Lizhen He*, Tianfeng Chen*

# R. Qiao and Z. Yuan contributed equally to this work.

* Corresponding Authors

College of Chemistry and Materials Science, Department of Oncology of The First Affiliated Hospital, Jinan University, Guangzhou 510632, China

E-mail: hlz6371@jnu.edu.cn (L. He), tchentf@jnu.edu.cn (T. Chen).

Supporting Figures

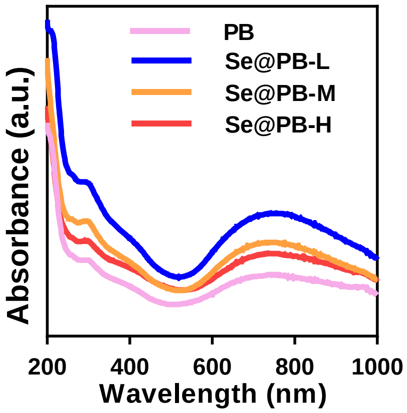

Figure S1. UV-Vis spectra of Se@PB nanoplatform with different Se content.

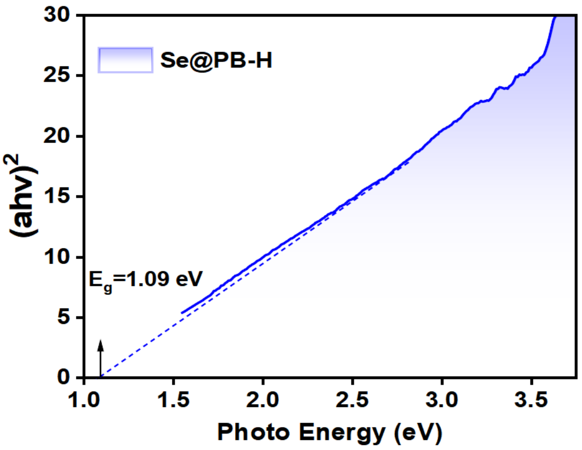

Figure S2. Bandgaps of Se@PB-H NPs obtained by UV−vis DRS and the Kubelka−Munk formula.

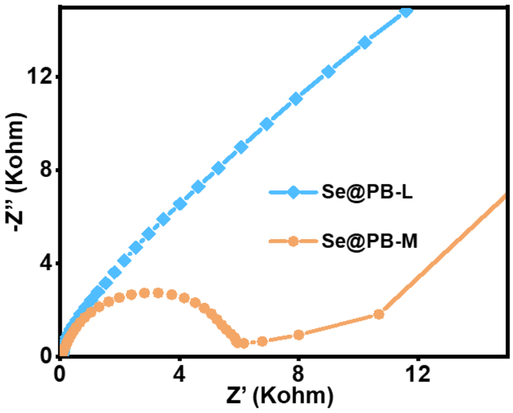

Figure S3. Impedance spectra of Se@PB-L and Se@PB-M.

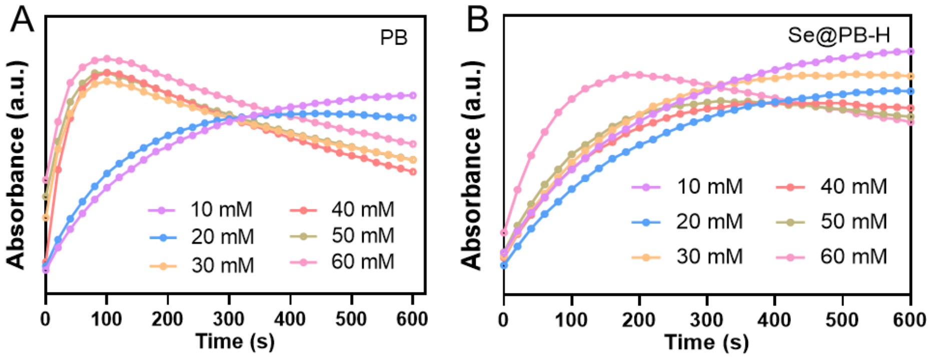

Figure S4. The catalytic processes of A) PB and B) Se@PB-H nano-heterojunctions at different concentrations of H_2O_2 were monitored by UV-Vis spectroscopy.

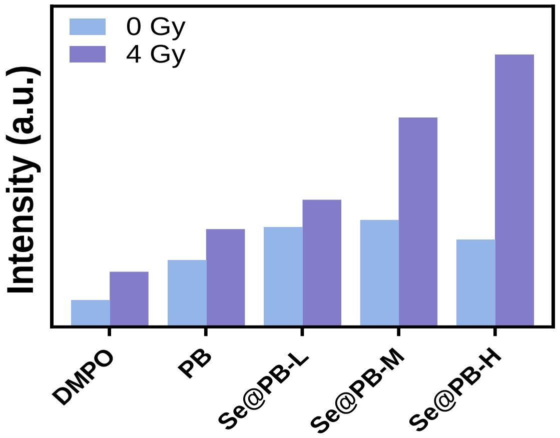

Figure S5. Quantification of ·OH level.

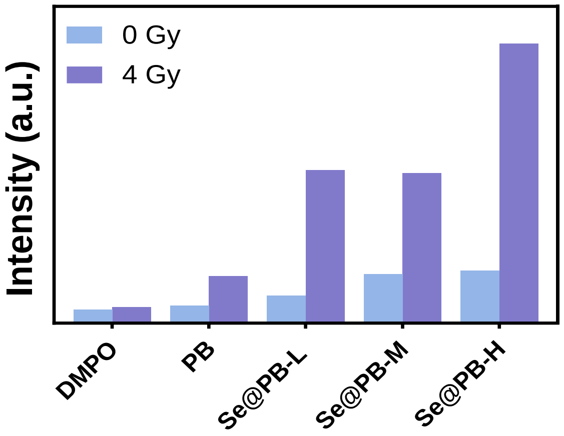

Figure S6. Quantification of $\cdot O_2^-$ level.

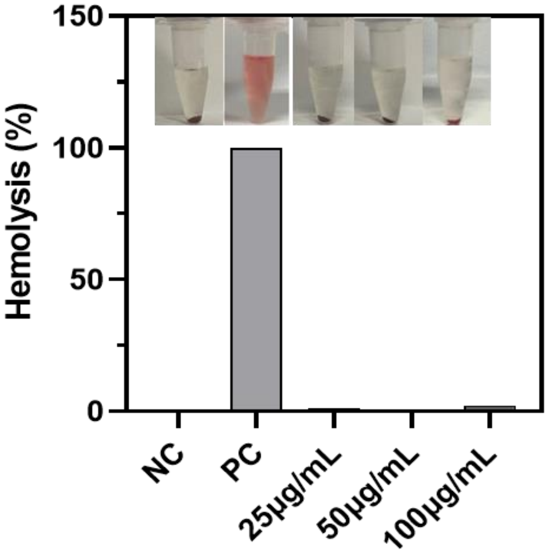

Figure S7. Biocompatibility analysis of Se@PB-H (R) at different concentrations. Where NC and PC denote negative and positive controls, respectively.

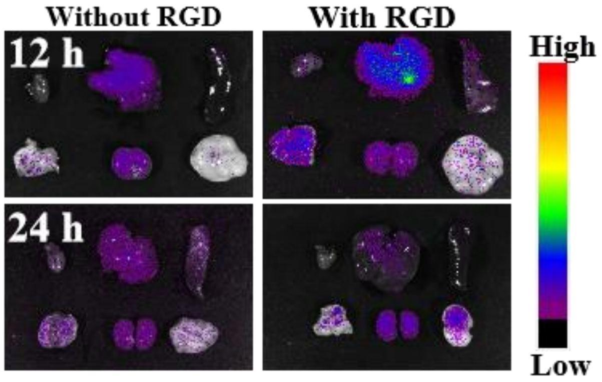

Figure S8. Fluorescence imaging of main organs of mice.

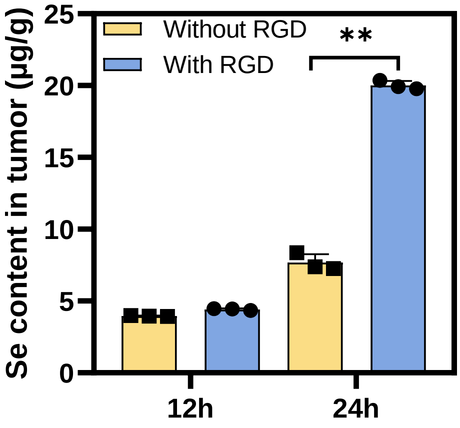

Figure S9. Se content in tumor tissue obtained from 12/24 h post intravenous injection (n = 3).

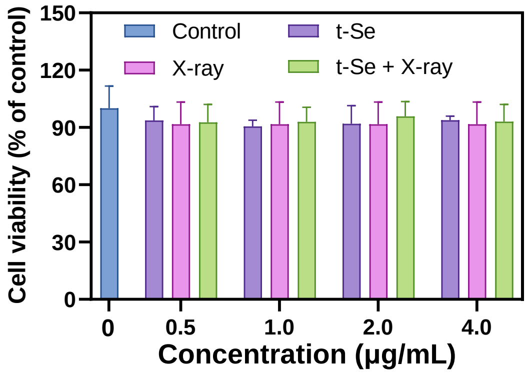

Figure S10. Evaluation of antitumor activity of t-Se in the presence or absence of X-ray.

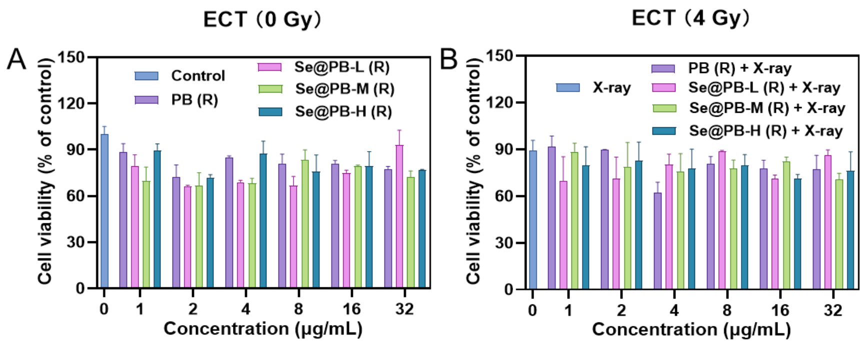

Figure S11. PB (R) and Se@PB (R) nanosystems-induced cytotoxicity in ECT cells at 72 h. A) without X-ray B) with X-ray of 4 Gy.

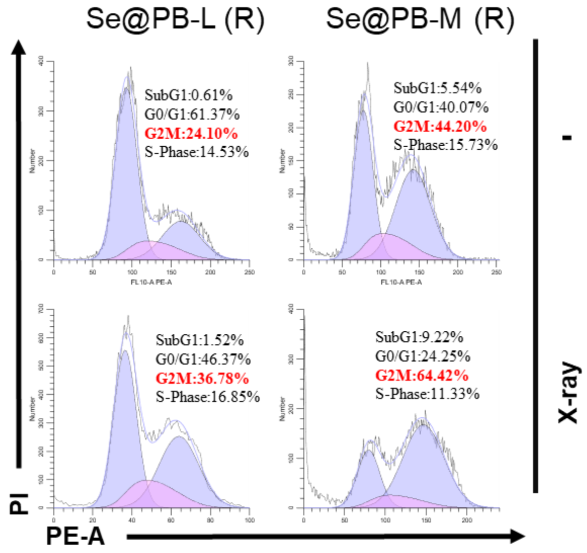

Figure S12. Cell cycle analysis when treated with Se@PB-L (R) and Se@PB-M (R) for 48 h with or without X-ray (4 Gy).

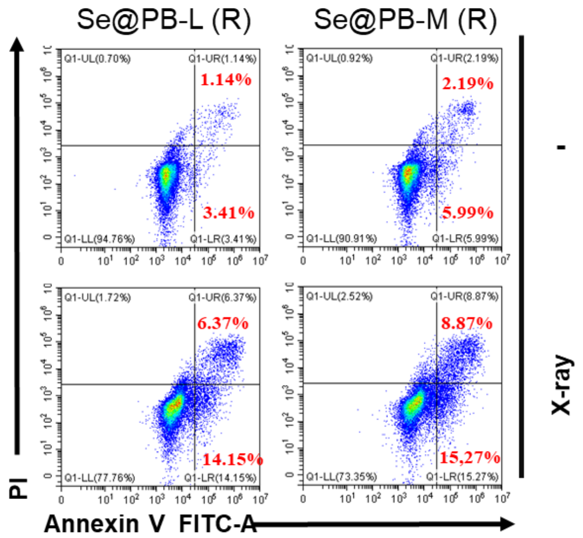

Figure S13. Cell apoptosis induced by Se@PB-L (R) or Se@PB-M (R) combined with/without X-ray (4 Gy).

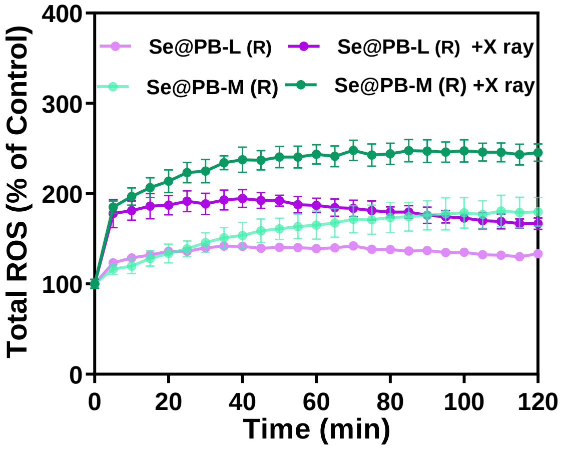

Figure S14. Levels of ROS production in HeLa cells by Se@PB-L (R) or Se@PB-M (R) (8 μg/mL) in the presence or absence of combined X-ray (4 Gy).

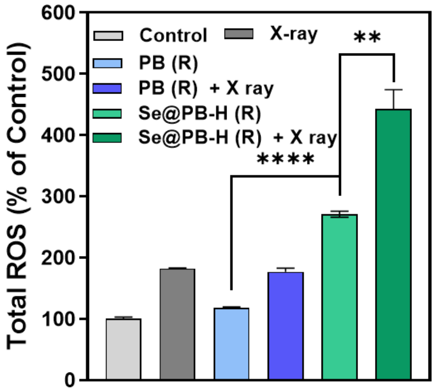

Figure S15.Quantification of DCF fluorescence intensity of different treatment groups.

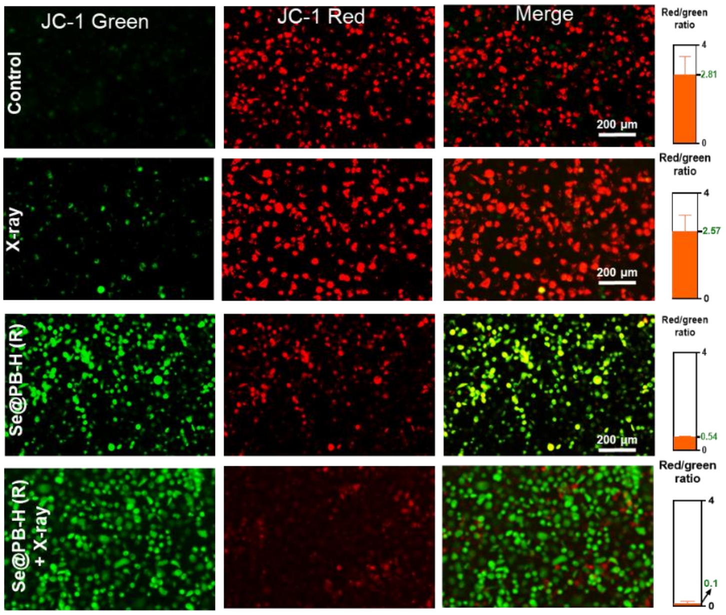

Figure S16. Mitochondria membrane potential assessed by JC-1 images of HeLa cells treated with Se@PB-H (R) and X-ray (4Gy).

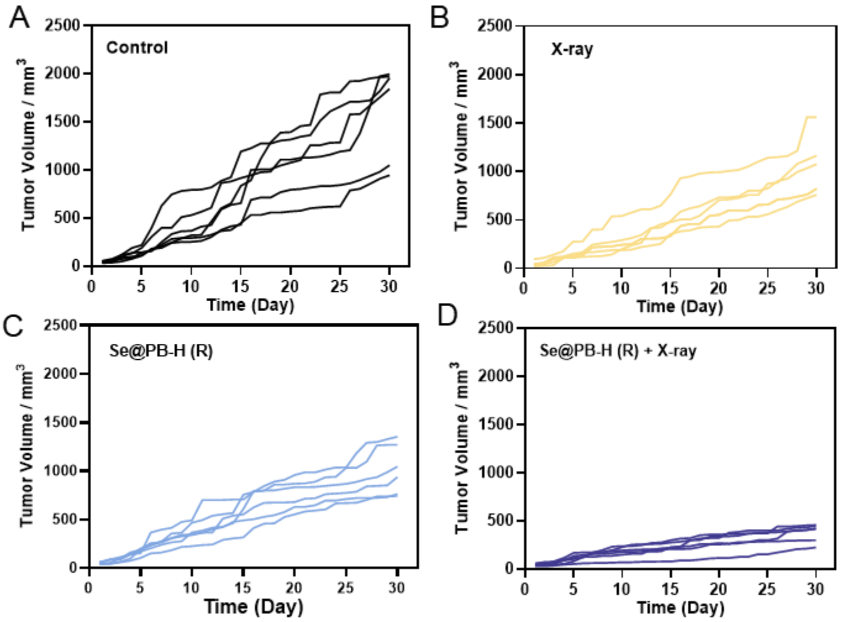

Figure S17. Curves of tumor volume of HeLa xenografts in nude mice after treatment with different groups. A) saline group, B) saline + X-ray group, C) Se@PB-H (R) group, and D) Se@PB-H (R) + X-ray group. (Se@PB-H (R) =5 mg/kg)

Table S1. Specific surface area, total pore volume, and mean pore size parameters for PB and Se@PB-H.

Sample	Specific surface area (m^2/g)	Total hole volume (mL/g)	Mean hole diameter (nm)
**PB**	88.4070	0.3777	17.0891
**Se@PB-H**	17.0891	0.2074	27.8848

Table S2. The sensitivity enhancement ratio (4 Gy) of radiosensitizers in this work and previous reports.

Radiosensitizer	Cell line	SER[a]	SI[b]	Reference
$MoSe_2$ NF-RGD	HeLa	1.69	/	[1]
$MoSe_2$ NF-RGD	SiHa	1.73	/	[1]
SeAuFe-EpC	MCF-7	1.83[a]	/	[2]
$Cu_{2-x}Se$	4 T1	1.1	/	[3]
$Cu_{2-x}Se$-Au	4 T1	1.6	/	[3]
Bi_2Se_3	A549	1.76	/	[4]
Bi/Se-Len NPs	Hep3B	2.36	/	[5]
Bi/Se-Len NPs	SMMC-7721	2.03	/	[5]
CS-Se	HeLa	1.6	/	[6]
CS-Se	SiHa	1.2	/	[6]
CS-Se	CaSki	1.1	/	[6]
Se@PB-L	HeLa	1.8	6.3	This work
Se@PB-M	HeLa	2.1	7.4	
Se@PB-H	HeLa	3.8	19.4	

a. Calculated by SER=IC_{50} (NPs)/IC_{50} (NPs + X-ray)

b. Safe Index (SI)= IC_{50}(normal cells)/IC_{50}(Cancer cells).

References

[1] W. X. Jiang, Z. Y. Zhang, M. M. Ye, S. Y. Pan, G. N. Huang, T. F. Chen, X. Q. Zhu, *Nano Today* **2022**, *46*.

[2] H. X. Liu, W. Q. Lin, L. Z. He, T. F. Chen, *Biomaterials* **2020**, *226*, 119545.

[3] Q. Huang, S. H. Zhang, H. Zhang, Y. B. Han, H. H. Liu, F. Ren, Q. Sun, Z. Li, M. Y. Gao, *Acs Nano* **2019**, *13*, 1342-1353.

[4] J. F. Xiao, L. J. Zeng, S. S. Ding, Y. M. Chen, X. Zhang, X. W. Bian, G. Tian, *Adv. Healthc. Mater.* **2022**, *11*, 2200143.

[5] J. N. Liu, J. Y. Chen, H. X. Liu, K. Zhang, Q. Zeng, S. Yang, Z. B. Jiang, X. T. Zhang, T. F. Chen, D. Li, H. Shan, *ACS Appl. Mater. Inter.* **2021**, *13*, 42473-42485.

[6] Y. C. Xu, H. Q. Lai , S. Y. Pan , L. L. Pan , T. Liu , Z. Y. Yang , T. F. Chen , X. Q. Zhu, *Biomaterials* **2024**, *305*, 122452
